# Employing electrochemically derived pH gradients for Lab-on-PCB protein preconcentration devices

**DOI:** 10.1038/s41378-023-00638-5

**Published:** 2024-01-18

**Authors:** Grace Maxted, Pedro Estrela, Despina Moschou

**Affiliations:** 1 Department of Electronic and Electrical Engineering, University of Bath, Bath, BA2 7AY UK; 2 Centre for Bioengineering and Biomedical Technologies (CBio), University of Bath, Bath, BA2 7AY UK

**Keywords:** Electrical and electronic engineering, Optics and photonics, Chemistry

## Abstract

Protein preconcentration is an essential sample preparation step for analysis in which the targeted proteins exist in low concentrations, such as bodily fluids, water, or wastewater. Nonetheless, very few practical implementations of miniaturized protein preconcentration devices have been demonstrated in practice, and even fewer have been integrated with other microanalytical steps. Existing approaches rely heavily on additional chemicals and reagents and introduce complexity to the overall assay. In this paper, we propose a novel miniaturized isoelectric focusing-based protein preconcentration screening device based on electrochemically derived pH gradients rather than existing chemical reagent approaches. In this way, we reduce the need for additional chemical reagents to zero while enabling device incorporation in a seamlessly integrated full protein analysis microsystem via Lab-on-PCB technology. We apply our previously presented Lab-on-PCB approach to quantitatively control the pH of a solution in the vicinity of planar electrodes using electrochemical acid generation through redox-active self-assembled monolayers. The presented device comprises a printed circuit board with an array of gold electrodes that were functionalized with 4-aminothiophenol; this formed a self-assembled monolayer that was electropolymerized to improve its electrochemical reversibility. Protein preconcentration was performed in two configurations. The first was open and needed the use of a holder to suspend a well of fluid above the electrodes; the second used microfluidic channels to enclose small volumes of fluid. Reported here are the resulting data for protein preconcentration in both these forms, with a quantitative concentration factor shown for the open form and qualitative proof shown for the microfluidic.

## Introduction

Diagnostic devices broadly include any devices that are used to identify the cause of an ailment. The scale of diagnostic devices varies from miniaturized biosensors, which can be as small as a few nanometers^1^, to magnetic resonance imaging (MRI), several square meters^2^, and can be stationary or portable. The benefits of portable devices are numerous. They enable healthcare professionals to take the test to the patient rather than transport the patient to a facility. This is especially important for those in an unstable condition or those with difficulty accessing hospital facilities. One such type of portable device is the point-of-care test, which allows testing to be performed in the community and outputs results in concordance with established laboratory methods^3^, an example of which is the COVID-19 lateral flow test, which has been so important in recent years^4–6^.

Point-of-care tests are required to be quick, inexpensive, readily available, easy to use and consume the minimum possible sample and reagent volumes^7^. Lab-on-a-chip (LoC) or micro total analysis systems (µTAS) are devices that shrink and automate processes ordinarily performed on a laboratory scale into devices of a few square centimetres^8^. The benefits of LoC are numerous, but of note are the decreased sample/reagent volume as well as speed, automation, and ease of use. Another key benefit is the potentially low cost of manufacturing a LoC device, which is especially important in low-/middle-income countries (LMICs), where the price of diagnostic devices is crucial.

Compared to conventional assays, LoC requires a significantly reduced quantity of sample; however, the potentially very low concentration of the target analyte in a low-volume (nano or microlitre scale) solution is an issue at both scales. To overcome this issue, it is possible to implement a method of preconcentration that increases the concentration of the target analyte. One such method is isoelectric focusing (IEF). This method uses a pH gradient to separate and focus charged amphoteric species according to their isoelectric point (pI) when an electric field is applied^9,10^. The pI is the pH at which the net charge of the molecule becomes neutral. IEF comes in many different forms, examples of which include free-flow isoelectric focusing (FF-IEF) and immobilized pH gradient gel isoelectric focusing (IPG-IEF)^11^, all of which depend on a change or gradient in pH.

Most methods for pH control consider bulk pH buffers, which do not allow the localized control that is desirable at low volumes. These buffers also require a number of chemical reagents that add complexity to practical implementations. A recently reported alternative approach to changing the pH of a solution is by introducing acid to the system through electrochemically generated acid (EGA). EGA is produced when a current or a voltage is applied to either a fluid containing chemicals such as quinones^12,13^ or to a surface on which chemicals have been immobilized^14^. This electronic control allows for greater quantitative control over the pH, and EGA is utilized here to induce a time-dependent change in pH on a printed circuit board (PCB) platform. This was produced with the goal of fabricating a microfluidic Lab-on-PCB device to quantitatively control pH to preconcentrate proteins within a sample. Such a development would allow, for the first time, the seamless integration of an electronic-based protein preconcentration module with an electrochemical protein biosensing module. This was achieved using commercial manufacturing technology, proving that mass-manufactured protein diagnostics for low-abundance biomarkers are ready for broad applications. As a significant step towards realizing such a device, in this work, we advance upon our previously published core technology^15^, utilizing it for the first time in a commercially produced PCB array to achieve protein preconcentration. Two case study proteins, bovine hemoglobin (bHb) and green fluorescent protein (eGFP), were used with optical and electrochemical methods to control and monitor the pH locally to an array of individually addressed electrodes.

## Results and discussion

**Fig. 1:**
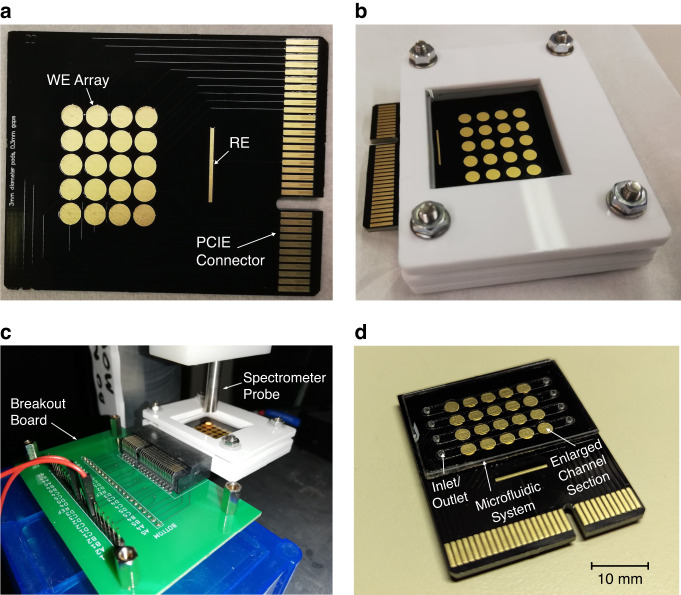
Images of the PCB platform in various configurations and setups. **a** 4 × 5 PCB array with 3 mm diameter electrodes spaced 0.3 mm apart. **b** PCB is in its ‘open air’ configuration in its holder and is capable of holding 1 mL of fluid for extended periods. **c** The ‘open air’ setup under the spectrometer while connected to its breakout board. **d** The PCB with its microfluidic system affixed

The device is shown in Fig. 1a and was used in two different forms: in its holder (Fig. 1b) and with integrated microfluidic channels (Fig. 1d). Before use; the PCB was cleaned using a series of electrochemical and chemical techniques, detailed in the Materials and Methods section, before being functionalized with 4-aminothiophenol (4-ATP). The 4-ATP self-assembled monolayer (SAM) was then electropolymerized using cyclic voltammetry (CV) between −0.2 and 0.7 V at 50 mV/s to improve the reversibility of the redox behavior of the SAM. After being fully processed, depending on the experiment conducted, the PCB was secured in one of its two forms. Its ‘open’ form included being secured in a holder and a uniform well of fluid suspended over the array, as shown in Fig. 1b, c. Its ‘closed’ form included a microfluidic channel, as shown in Fig. 1d. In its open setup, samples were able to be freely extracted throughout the experiment. However, in its microfluidic form, this physical removal of the sample was no longer possible, and all data collection was required to be performed without interrupting the system.

**Fig. 2:**
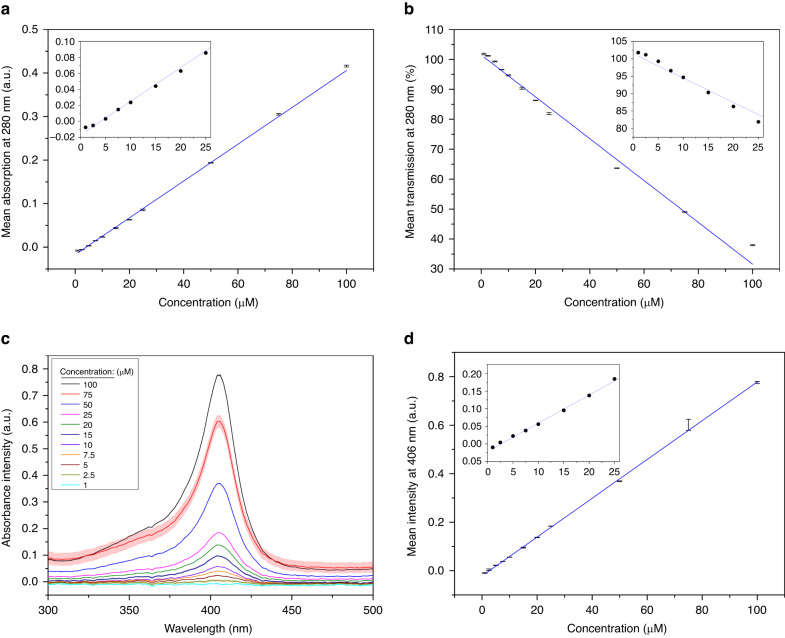
Various calibration methods for bHb in 10 mM PBS for concentrations between 1 and 100 µM. **a** absorbance at 280 nm; **b** transmission at 280 nm; **c** full spectrum of absorbance intensity; **d** mean intensity at 406 nm. With linear fits of *R*^2^ **a** 0.9989 **b** 0.98639 and **d** 0.99818

Before protein preconcentration trials using the PCB array in the open form, the optical response of different concentrations of bovine hemoglobin (bHb) was first calibrated between 100 µM and 1 µM. Initially, the absorption and transmission at 280 nm were measured using a nanovolume spectrometer; this was performed as this wavelength maps linearly to the maximum absorbance in proteins^16^. Figure 2a, b shows the results for the absorbance and transmission at 280 nm, respectively, and show the linear fits, which have *R*^2^ values of 0.9989 and 0.9864. Full spectrum analysis was also conducted using a nanovolume spectrometer to provide insight into the optical properties of the solution between 300 and 500 nm (Fig. 2c). This analysis displayed a clear peak at 406 nm, the intensity of which is shown in Fig. 2d, and has a strong linear relationship with the protein concentration (*R*^2^ value of 0.9982). The importance of these data lies in the ability of the full spectrometer system to record this wavelength accurately; this is because 280 nm falls outside of its working range of 350–880 nm and thus had little use when the device was sealed into its microfluidic form.

**Fig. 3:**
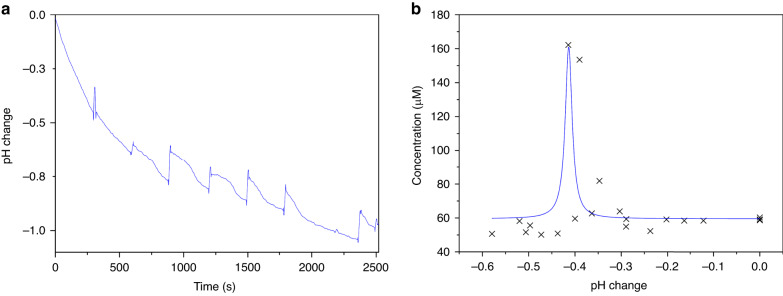
Data showing the results for open air protein preconcentration using bHb. **a** pH change over time when samples were physically extracted; **b** pH change against concentration of bHb

For the open configuration of the device, where the extraction of physical samples was still possible, the absorption at 280 nm was chosen as the calibration method due to its slightly higher *R*^2^ value. Preliminary experiments showed that physically extracting 2 µL samples should be performed more than 2 min apart to minimize the effect on the environment; 5 min was selected, and the pH response over time when 0.4 V CA was applied as shown in Fig. 3a. Samples were taken from above the pad on the array, which was connected as the working electrode (WE), and transferred to the nanovolume spectrometer for analysis. Between uses, the machine receptacle was cleaned with ultrapure water (Milli-Q) and dried with fiberless tissue.

The PCB was cleaned, functionalized, and electropolymerized before being secured into its open setup (see Fig. 1c). A solution of 50 µM bHb and 100 µM 5(6)-carboxynaphthofluorescein (CNF, Sigma-Aldrich, United Kingdom) was prepared in 10 mM phosphate-buffered saline (PBS, Sigma-Aldrich, United Kingdom) (pH 7.1 ± 0.1), 1 mL of which was pipetted into the well to create a well with a uniform path length of 1.4 mm. This step served to improve the consistency of the data collected using the USB spectrometer; however, it was noted that the surface tension at the edges of the PCB holder caused imperfections in the uniform path length that the holder was designed to create. Then, 0.4 V was applied to the WE, while a gold pseudoreference on the PCB was used as the counter electrode (CE)/reference electrode (RE).

Before the potential was applied, a 2 µL sample was taken from above the WE and analyzed with a nanovolume spectrometer. Then, 0.4 V chronoamperometry (CA) was applied to the WE for 30 min, and a 2 µL sample was collected from just above the electrode surface every 5 min. The results for this are shown in Fig. 3b and show a peak at a pH change of −0.4; this corresponds to a pH of 6.7 ± 0.1, where 6.8 is the pI of bHb^17^, and a concentration factor (CF) of over 3. This figure also demonstrates some of the expected behavior of an ideal protein preconcentration experiment, showing a variation of a Gaussian distribution with a flat response on either side of a single peak. With the device working in its open configuration, microfluidics were subsequently designed using *AutoCAD 2022* (Autodesk, United States) to affix to the PCB array, as shown in Fig. 1d.

The two forms of the device present distinct advantages and disadvantages. As previously mentioned, the ability to extract samples from the open device for external analysis allows for confidence in the technique to be established and additional tests to be run. However, due to the relatively large volume of fluid, the pH gradient formed through EGA must be thought of as spreading not only horizontally across the array but also vertically between the WE and the spectrometer probe. This requires that any pH reading from the USB spectrometer must be taken as the average pH across the path length of the beam and not as an absolute value. In addition, the volume of sample needed is far larger, which diminishes some of the advantages of the device concept. These issues are directly countered by the introduction of microfluidics to the device, as the depth of fluid above the WE is significantly less than that of the open device in its holder. The microfluidic channels allow for greater confidence in the absolute value of the pH above the WE and allow for more control in the formation of a pH gradient. Microfluidics, however, have their own inherent disadvantages. Bubbles within channels can cause significant problems with flow as well as with electrochemical processes. Sample extraction also becomes more complex than that of an open device. Furthermore, sophisticated channel design and valves may also be required to tailor the flow, which would require further development and optimization.

**Fig. 4:**
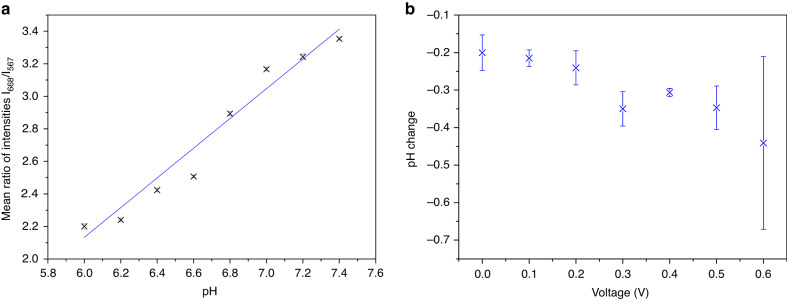
Data illustrating the final characterization steps for CNF in the mircorfluidic PCB system. **a** Calibration of 1 mM CNF in pH buffers between pH 6.0 and 7.4, linear fit with *R*^2^ value of 0.96144; **b** pH change results for 20 min CA for potentials between 0.1 and 0.6 V

**Fig. 5:**
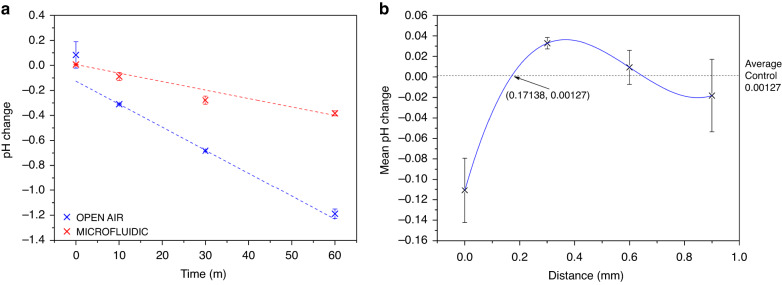
Data showing the comparison and characterisation of the microfluidic system compared to the open air configuration. **a** pH change over 60 min with linear fits, *R*^2^ value of 0.95994 for the open air and 0.91439 for the microfluidic configuration; **b** pH change recorded for the microfluidic setup at different distances from the WE, showing a spatial resolution of 0.17138 mm

Preliminary data with this microfluidic device showed issues when measuring low concentrations of CNF, as the number of molecules was insufficient to measure the pH optically. The concentration of CNF was increased tenfold to 1 mM in 10 mM PBS and recalibrated in a more refined pH range, pH 6.0–7.4, the results for which are shown in Fig. 4a. CA of different voltages were evaluated for 20 min to confirm whether 0.4 V remained the optimal potential for EGA; potentials between 0.1–0.6 V were tested. These results are shown in Fig. 4b and clearly illustrate that 0.4 V gives the most reliable change in pH, −0.31 ± 0.01, over a period of 20 min. While 0.3, 0.5, and 0.6 V drive a higher pH change, the reliability is low compared to that of 0.4 V, and thus these conditions were not reused. The error in the measurement of 0.6 V was especially high, and it was determined that at the potential, the electrodes were unstable, with some burnout having been observed. The WE and CE/RE components were pads within the same channel, and these channels were designed such that minimal optical scattering would occur with the incorporated microfluidic layers. The EGA was then performed for 60 min for direct comparison between the open and microfluidic systems, which is shown in Fig. 5a. Despite showing a smaller response for the pH change in its microfluidic form, there is a clear, linear decrease in pH shown, and the system was further characterized.

**Fig. 6:**
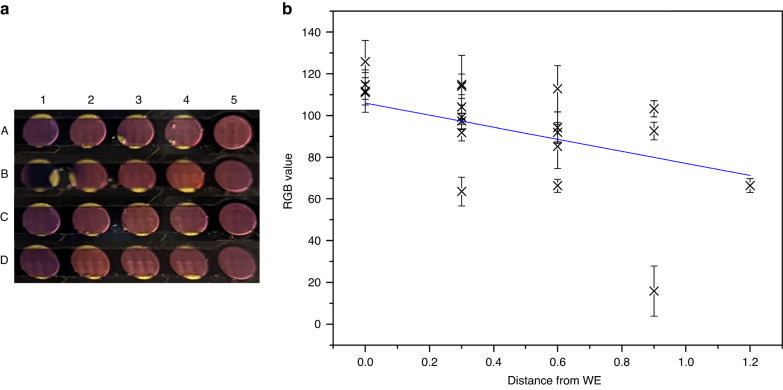
Optical analysis of the electrochemical generation of acid in the microfluidic PCB systemn using RGB methods. **a** Image of the microfluidic channels when filled with 1 mM CNF and 0.4 V CA applied for 20 min; from top to bottom, the pad used as the WE shifts from A5 to D2; **b** red (R) value from RGB analysis at different distances from the WE

The behavior of the microfluidic device was further characterized by defining the spatial resolution of the microfluidic systems, which we have defined in our published work^18^ as “*the distance at which the effects of EGA are no longer observed via our optical characterization method, using our control value as the benchmark threshold*”. The detailed procedure for how this was achieved may also be found in that work^18^. Here, a CA of 0.4 V was applied for 20 min to pads in the microfluidic channels at different distances from the spectrometers’ position. In the nonmicrofluidic form, the spatial resolution was found to be 0.664 mm, while in its microfluidic form, we report that this property is 0.171 mm, which is shown in Fig. 5b. Therefore, all data on our array with a spacing of 0.9 mm may be considered independent. The overlap in the error bars for 0.6 and 0.9 mm supports this statement, as it would be expected that above the spatial resolution, the data should be effectively the same. This experiment also yielded visual data, which is shown in Fig. 6a; from top to bottom, the pad used as the WE was changed from A5 to D2, while the optical analysis was held steady in Column 5 throughout. CNF is known to change color as the pH shifts from basic (blue) to neutral (purple) to acidic (pink/red). This, coupled with the RGB analysis of the image, Fig. 6b, shows that there is a clear pH gradient formed in the channel when EGA is performed, which was the prerequisite for performing microfluidic IEF (µIEF).

**Fig. 7:**
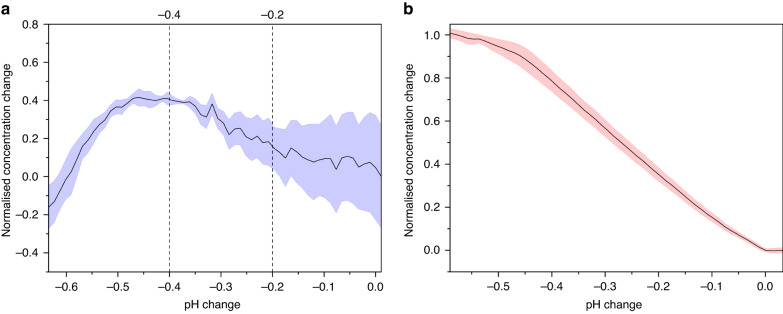
Protein preconcentration results for the microfluidic PCB system using two different proteins. **a** bHb, graph of average normalized intensity at 406 nm against pH change; **b** eGFP, graph of average normalized intensity at 507 nm

To perform protein preconcentration using µIEF on the individually addressed PCB array, PCBs were first prepared. They were stripped of thiols, cleaned using SC-1, functionalized with 4-ATP for more than 19 h to form a well-ordered SAM, electropolymerized, as before, and the microfluidic ‘sandwich’ secured atop the array. For bHb, a solution of 50 µM bHb and 1 mM CNF was prepared in 10 mM PBS (pH 7.1 ± 0.1), and 0.4 V CA was applied to the WE for 30 min to achieve a pH change of −0.58 ± 0.03. A 200-point Savitzky‒Golay (SG) smoothing filter was applied, and the pH change was compared against the normalized intensity at 406 nm. This method for assessing the concentration of bHb was used, as it was not possible to physically extract a sample from the sealed channels. As the concentration is directly correlated to the absorption intensity at this wavelength, as shown in Fig. 2d, and the substrate is optically thick, the change in concentration over time may be equated to the change in reflective intensity over time. These data are shown in Fig. 7a and show the averaged data and the standard error of the mean, along with the range in which a peak would be expected to lie, corresponding to 6.8 ± 0.1. Qualitatively, the intensity at 406 nm, which directly correlates to the concentration of bHb, changes with the pH change and shows a clear peak at a pH change of approximately −0.4. This corresponds to a pH of 6.7 ± 0.1, which, as the pI of bHb is pH 6.8, clearly demonstrates that the device is capable of concentrating protein samples.

eGFP was then assessed using a process that only differed from that used for bHb by changing the protein and the concentration of that protein from 50 µM to 25 µg/mL, which equates to approximately 929 nM. As the pI of eGFP is pH 6.2^19^, it was not expected that with a pH change of approximately −0.6, a complete peak would be observed, which is confirmed by the data shown in Fig. 7b. This graph shows the normalized intensity at 507 nm, which is the emission wavelength for this protein, and shows a clear increase in intensity as the pH decreases. However, unlike bHb, this wavelength falls significantly closer to the emission wavelength for CNF in acidic/neutral pH, 567 nm, which may impact the reliability of these data.

## Materials and methods

As per our previously published work^18^, 4-aminothiophenol (4-ATP, Sigma-Aldrich, United Kingdom) was used to functionalize the surface of gold electrodes on PCB arrays with a self-assembled monolayer (SAM). These PCBs were designed in *Altium Designer* and manufactured by Newbury Electronics Ltd. (United Kingdom). Figure 1a shows the 4 ×5 PCB array of 3 mm diameter electrodes with a spacing of 0.3 mm; however, for the data presented here, the array spacing was 0.9 mm. A holder was designed for the open-air experiments (see Fig. 1b), and the PCB itself was designed to connect using a 64-pin PCIE connector. This connector was mounted into a breakout board (see Fig. 1c), which allowed for theoretical simultaneous control over all pads in the array.

The PCBs were first cleaned by wiping them with acetone, isopropanol (IPA), and ultrapure water (Milli-Q) and then dried with nitrogen. Any existing thiols on the surface were then stripped using CV in 100 mM NaOH (Sigma-Aldrich, United Kingdom) for 50 cycles at 1 V/s between −1.5 V and 0.5 V using an external platinum CE and Ag/AgCl reference electrode (RE). This was then followed by 15 min of immersion in a 5:1:1 solution of Milli-Q, 30% hydrogen peroxide (Sigma-Aldrich, United Kingdom), and ammonium hydroxide (Sigma-Aldrich, United Kingdom), after which the board was rinsed with Milli-Q. The PCBs were sequentially immersed for 5 min in acetone, isopropanol, and Milli-Q and dried with nitrogen; this process is a standard clean (SC-1) as defined in the literature^20^. After cleaning, the PCBs were immediately immersed in 0.5 mM 4-ATP dissolved in absolute ethanol to functionalize overnight for a minimum of 19 h to form a well-ordered SAM. This time period was experimentally determined to produce a stable SAM and is consistent with the literature on the subject^21^.

After functionalization, the chips were rinsed with absolute ethanol and Milli-Q and dried with nitrogen before being electrochemically polymerized. CV between −0.2 and 0.7 V at 50 mV/s was performed using a platinum CE and Ag/AgCl RE for three cycles in 10 mM phosphate-buffered saline (PBS, Sigma-Aldrich, United Kingdom) to polymerize the 4-ATP SAM, forming head-to-toe dimers. 4-ATP in the form of a SAM will exchange protons with a solution when exposed to voltage^21^, the reversibility of which may be improved upon polymerization^21,22^. In electrochemistry, reversibility describes the rate of electron transfer^23^. In its unpolymerized form, the redox reaction of a 4-ATP SAM may be described as irreversible, where the rate of reaction is slow, whereas, in its polymerized state, the rate of reaction is increased and may be classed as quasireversible^22^.

For the microfluidic experiments, a stack of 130 µm thick double-sided adhesive (468 MP, 200 MP adhesive, 3 M, United States) was created with 50 µm thick poly(methyl methacrylate) (PMMA) film and laser cut to form channels capable of holding approximately 21 µL of fluid. The channels were sealed with a 1 mm thick PMMA lid that was laser cut to size, and holes were micromachined to fit 20-gauge dispensing tips (RS Components, United Kingdom). These layers were then secured to the PCB and clamped under uniform pressure to seal the channels; fiberless wipes were used to protect the surfaces of the device during this process.

To measure and monitor the pH local to each individually addressed electrode, a pH-sensitive dye, 5(6)-carboxynaphthofluorescein (CNF, Sigma-Aldrich, United Kingdom), was used alongside a USB spectrometer. CNF is particularly sensitive in the range of pH 6–8^24,25^ and fluoresces at a wavelength of 668 nm in basic solutions and 567 nm in acidic and neutral solutions^24^. The ratio of the intensities at these wavelengths was used to characterize the dye. Under basic conditions, fluorescein will undergo a structural rearrangement that changes the molecules’ optical properties; fluorescein changes between its open and closed forms at low and high pH, respectively^26^. Bovine hemoglobin (bHb, Sigma-Aldrich, United Kingdom) and fluorescent green protein (eGFP, Chromotek, United States) were selected due to their isoelectric points (pIs) of pH 6.8 and 6.2, respectively, which are ideally situated in the midst of the working range of CNF. Initial calibration of the behavior of 100 µM CNF was achieved by using a range of buffers of known pH from pH 4 to 10 and taking the ratio of intensities at 668 and 567 nm. Linear behavior between pH 6 and 9 was observed, and functionality between pH 6–8 was verified. Later, calibration for 1 mM CNF was performed between pH 6.0 and pH 7.4.

The USB spectrometer used was configured for the range of 200 nm < *λ* < 880 nm and was used in conjunction with a long-life tungsten source typically used in a wavelength range of 350–1700 nm, limiting the working wavelength range for the optical setup to 350–880 nm. The software *OceanView* (OceanInsight, United States) was used to record the spectroscopic data and was used to perform preprocessing of the data, which consisted of a Boxcar filter and averaging the data over 10 scans. The Boxcar filter was used to half the signal-to-noise ratio (SNR) by averaging over 4 data points^27^. *OriginLab* was used as the primary method for data analysis, fitting, and postprocessing, and depending on the data and method, a smoothing low-pass filter, SG^28^, was used to reduce the impact of noise. The EGA on a 4 ×5 electrode array on a commercially manufactured PCB was controlled using potentiostats (Palmsens 4, Ivium Compactstat, Ivium Pocketstat) and was continuously monitored optically. Chronoamperometry (CA) was used in a 2-electrode setup with a gold-pseudo reference/counter electrode. 0.4 V was continuously applied to the functionalized and polymerized electrodes, a detailed explanation of why CA of 0.4 V was initially selected over 0.2 V and 0.3 V as shown in our previous paper; this voltage provides an approximately linear decrease in pH^18^.

A variety of spectroscopic methods were utilized to characterize and measure the preconcentration response of the two proteins used. While in the open-air configuration, 2 µL samples were physically extracted using a pipette and transferred to the Genova Nano Microvolume Spectrophotometer (GNMSpec, Jenway, United Kingdom), where they underwent spectroscopic analysis. When the PCB was in its microfluidic form, as shown in Fig. 1d, the aforementioned USB spectrometer setup was used to measure fluorescence in the sealed channels.

Within the channels, absorption data were not possible to collect with the USB spectrometer; however, reflection data were. In spectrometry, there are three ways in which light behaves when it is in contact with the substrate: reflected, absorbed, or transmitted. Because the substrate is optically thick, it is assumed that there is no transmission of light and that the absorbed and reflective light may, therefore, be treated as interchangeable for the purpose of qualitatively determining the way in which the concentration of proteins within the channel changes. Throughout the protein preconcentration experiment, the emission wavelengths of bHb and eGFP were measured over time.

## Conclusion

In this work, we present the first isoelectric-focusing-based protein preconcentration Lab-on-Chip based on an electrochemically derived pH gradient. This device incorporates of our previously reported Lab-on-PCB device to control the pH of a solution through electrochemical generation of acid using a 4 ×5 gold electrode array. Electrodes with a diameter of 3 mm and spacing of 0.9 mm were used in two configurations, with one open to the air and the other enclosed in microfluidic channels capable of holding approximately 21 µL. Gold working and pseudoreference electrodes were used in a 2-electrode configuration to electrochemically control the pH of a solution using CV and CA, a USB spectrometer was used in conjunction with a fluorescent dye to measure the pH of the solution and a nanovolume spectrometer was used to measure protein concentrations.

The electrodes were cleaned, stripped, and subsequently functionalized with a thiol-terminated molecule to form a SAM, which was electrochemically polymerized using CV to improve the electrochemical reversibility. Potentials were applied to one pad in the array, which served as the WE, and to another pad in the array, which served as a gold pseudo-reference electrode. A CA of 0.4 V was applied for a range of time periods to characterize the device and induce a change in the pH of a solution comprising mostly 10 mM PBS.

In open-air configuration, a clear peak was observed in the optically measured concentration of the protein, bHb, at the pH corresponding to the protein’s pI and a concentration factor of more than 3. Microfluidic channels were subsequently designed and formed using layers of double-sided adhesive and one thin and one thick layer of PMMA. These channels were successfully used to qualitatively prove that the device was capable of preconcentrating proteins when a voltage of 0.4 V was applied over a period of 30 min. This demonstrates the devices’ potential for integration into existing LoC or Lab-on-PCB devices where the target analyte has an inherent net charge.

We propose that our approach of employing electrochemistry, rather than chemical reagents, can simplify isoelectric focusing devices and novelly advance them toward use as practical components in low-abundance protein quantification Lab-on-Chip microsystems. The practical implementation we present here via Lab-on-PCB further enables the incorporation of such devices as components in high-complexity, seamlessly integrated microsystems.
